# Effects of the birthing room environment on vaginal births and client-centred outcomes for women at term planning a vaginal birth: BE-UP, a multicentre randomised controlled trial

**DOI:** 10.1186/s13063-018-2979-7

**Published:** 2018-11-19

**Authors:** Gertrud M. Ayerle, Rainhild Schäfers, Elke Mattern, Sabine Striebich, Burkhard Haastert, Markus Vomhof, Andrea Icks, Yvonne Ronniger, Gregor Seliger

**Affiliations:** 1 Institute of Health and Nursing Science, Medical Faculty of the Martin Luther University Halle-Wittenberg, Magdeburger Str. 8, 06112 Halle (Saale), Germany; 2 Department of Health Science, Hochschule für Gesundheit - University of Applied Sciences, Gesundheitscampus 6-8, 44801 Bochum, Germany; 3 mediStatistica, Neuenrade, Germany; 4 Institut für Versorgungsforschung und Gesundheitsökonomie, Centre for Health and Society/ German Diabetes Centre, Medical Faculty of the Heinrich Heine University, Moorenstraße 5 / Geb. 12.49, 40225 Düsseldorf, Germany; 5 Coordinating Centre for Clinical Trials (KKS) Halle, Medical Faculty of the Martin Luther University Halle-Wittenberg, 06097 Halle (Saale), Germany; 6 University Hospital and Polyclinic of Obstetrics and Prenatal Medicine, Medical Faculty of the Martin Luther University Halle-Wittenberg, Ernst-Grube-Straße 40, 06120 Halle (Saale), Germany

**Keywords:** Vaginal birth, Birth environment, Self-determination, Maternal satisfaction, Midwifery, Hospital setting, RCT, Health economic evaluation

## Abstract

**Background:**

Caesarean sections (CSs) are associated with increased risk for maternal morbidity and mortality. The recommendations of the recently published German national health goal ‘Health in Childbirth’ (*Gesundheit rund um die Geburt*) promote vaginal births (VBs).

This randomised controlled trial (RCT) evaluates the effects of a complex intervention pertaining to the birth environment, based on the sociology of technical artefacts and symbolic interactionism. The intervention is intended to foster an upright position and mobility during labour, which lead to a higher probability of VB.

**Methods/design:**

This study is an active controlled superiority trial with a two-arm parallel design. The complex intervention involves making changes to the birthing room to encourage an upright position and mobility of women in labour and to relax them, which may help them to cope with labour and may increase self-determination. This may result in more VBs. Included in the study are primiparae and multiparae with a singleton foetus in cephalic presentation at term planning a VB. According to the sample size calculation, 3800 women in 12 obstetrical units are to be included. Randomisation will be performed centrally and controlled by an independent coordination centre. Blinding of participants and staff is not possible. Key outcomes are VB, episiotomy, perineal tears, epidural analgesia, critical outcome of newborn at term and maternal self-determination during birth. Additionally, a health economic evaluation will be performed.

**Discussion:**

This is the first adequately powered multicentre RCT examining the effect of a redesigned birthing room on the probability of a VB and patient-centred physical and emotional outcomes. An increase in the number of VBs by 5% from a baseline of 74% to 79% would result in 21,000 women per year experiencing a VB rather than a CS in Germany. Expected benefits are greater self-determination during labour, improved physical and emotional client-centred outcomes, fewer medical interventions and a reduction in health-care costs.

**Trial registration:**

German Clinical Trials Register (*Deutsches Register Klinischer Studien*), DRKS00012854. Registered on 7 March 2018.

**Electronic supplementary material:**

The online version of this article (10.1186/s13063-018-2979-7) contains supplementary material, which is available to authorized users.

## Background

### Mode of birth

The rate of caesarean sections (CSs) in Germany in 2016 [1] was higher than recommended by Betran et al. [2] despite being associated with an increased risk for maternal morbidity and mortality [3]. The average CS rate was 32.1%, with regional differences ranging from 17% to 51% [1]. Of these, 18.7% were repeat CSs with no additional medical indication whilst 30.7% had additional medical indications [1].

Of all live hospital births, 80.6% (*n* = 623,685) were singleton births at term in cephalic presentation. Of these, 75.2% (*n* = 469,195) were spontaneous vaginal births (VBs) or operative vaginal births (OVBs) [1]. In the federal states of North Rhine-Westphalia and Saxony-Anhalt in 2016, VB and OVB rates were 73.1% and 75.0%, respectively [4, 5].

In Germany, most spontaneous VBs occur in a maternal recumbent position on a birthing bed. In 2016, there were 77,123 per 100,000. Only 9616 per 100,000 are documented to have occurred in an upright or unspecified position [1]. In international studies, low-risk women in recumbent positions in the first stage of labour were more likely to have an epidural, a CS or their babies admitted to a neonatal intensive care unit [6]. Recumbent positions in the second stage of labour are associated with increased administration of opioids and oxytocin [7], a higher rate of episiotomies [7] and vaginal operative births [7, 8], and increased levels of pain [7, 8] and fatigue [9, 10].

If labour is prolonged or obstructed, there appears to be room for behavioural interventions to increase the possibility of a VB. The prevalence of prolonged or obstructed labour in Germany in 2016 was 6.5% (sole diagnosis) or 21.1% if documented alongside other diagnoses [1].

### Interventions to increase vaginal births

A literature review was conducted via a search of MEDLINE (via Ovid and PubMed), Cochrane Central, the Cochrane library, clinicaltrials.gov, Deutsches Register Klinischer Studien, International Clinical Trials Registry Platform, EMBASE, PSYNDEXplus, CINAHL and MIDIRS using the medical subject headings ‘delivery room’, ‘hospital design and construction’, ‘birth environment’ (plus all combinations of ‘unit’, ‘room’, ‘bed’ or ‘space’), ‘vaginal birth’ and ‘low risk’. No time limits were set. Quantitative and qualitative designs of high quality were sought, most recently on 28 October 2017. One systematic review [11] and five Cochrane reviews [6, 12–15] were retrieved. Additionally, three randomised controlled trials (RCTs) [16–18], a cohort study [19], a pilot study [20], a number of relevant qualitative studies [21–28] and a theoretical paper [29] were found. The findings are summarised below.

- Upright maternal positions and mobility (vs. conventional care) in the first [6] and second stages of labour [13, 15]: Interventions in position and mobility in the first stage of labour lessened the likelihood of an epidural and a CS [6]. In the second stage of labour, they reduced the incidence of abnormal foetal heart rate patterns, episiotomies and OVBs. However, the trials were found to be methodically weak [6, 13] and the data inconclusive [15]. In particular, evidence is lacking regarding effects on perineal tears, increased blood loss [13] and neonatal outcomes [6, 13].
- Environmental interventions in health-care settings: The systematic review found that such interventions had a (small) positive effect on women’s ability to cope with pain and their personal interactions [11]. In the single cohort study [19], a redesigned single-room maternity care unit (*n* = 250) was compared with a traditional delivery care unit (historical comparison group). The quality of maternity care, maternal comfort and coping with pain, and respect for privacy were reported to be significantly improved. The study did not assess VBs as an outcome and the trial was conducted 17 years ago.
- Alternative or home-like (vs. conventional) institutional settings: An alternative or home-like design increased VB rates, satisfaction with care and initiation of breastfeeding. The likelihood of oxytocin augmentation in labour, intrapartum analgesia or anaesthesia, episiotomy and OVBs decreased. However, the reviewers found it ‘difficult to draw inferences about the *independent effects of the physical birth environment*’ due to the different organisational models of care of the alternative birth settings studied [14].
- Redesign of the birthing room: There are indications that redesigning the birthing room might have a positive effect on VB rates [14]; however, statistical power was lacking in the two relevant studies [19, 23]. In two single-centre RCTs, nature scenes on a television [16] and calming audio-visual stimuli [17] during labour had positive effects on the perceived quality of care, 5-minute Apgar scores [16] and pain intensity [17].

A single-centre trial in Denmark, which was due to be completed in April 2018, studied the effect of a redesigned birthing room on oxytocin augmentation during labour [18]. The results are not yet available. The PLACE pilot trial, which specifically examined the effect of the immediate birthing environment (ambient birthing room) on VBs was evaluated positively by women and staff [20].

Based on the available literature, Foureur et al. [29] developed a hypothetical model of a ‘safe satisfying birth’, in which the model of care and the design of the birth unit are basic environmental dimensions that encompass the stress experienced by women in labour and by staff, and their communications with each other.

Qualitative research focussing on the birth environment highlights various interconnected influences: women in labour experienced subjective relaxation, comfort and a sense of control in a *Snoezelen room* [24], whereas in a traditional delivery room they felt as though they were ‘objects of surveillance’ [27]. The role of support people is hampered in birthing rooms due to limited space, lack of equipment to support the woman, and lack of places to rest. These factors restrict their endeavours to support women in labour; they themselves feel ‘in the way’ and that their needs are disregarded [23].

In particular, women in labour expect continuity of care, choice and control [25]. They also expect emotional support from their partner or companion, and that midwives will facilitate their active involvement in care [26]. A study focussing on birthing beds revealed that in developed countries, the birthing bed is culturally important in terms of the safety of maternity care. Encouraging women to stay off the bed is associated with defying the norm [28]. In a hospital, the design of a birthing room and its equipment should not negatively impact on midwifery practice [21], but rather convey a sense of ‘friendliness, functionality and freedom’ and allow care to be tailored to the needs of each woman [22].

### Need for reliable evidence

In summary, as yet there is no conclusive evidence regarding the independent effect of the birthing environment. Changes to the birth environment to facilitate maternal mobility are required to encourage physiological births [30] and thereby positively influence women’s experiences and the care provided by staff. In terms of clients’ rights, it is essential that women are offered more choices for self-determination during labour and birth.

International guidelines from Europe and Australia [31, 32] and non-governmental organisations (such as the Mother-Friendly Childbirth Initiative [33]) highlight the importance of introducing measures to support women in their aim to experience a VB. The recently published German national health goal ‘Health in Childbirth’ (*Gesundheit rund um die Geburt*) [34] is also intended to foster physiological births.

Given the lack of evidence, an adequately powered RCT is required to provide more robust results, which would be nationally and internationally informative. We have, therefore, decided to conduct the multicentre active controlled superiority trial outlined below. The research question is: Does a redesigned birthing room that fosters maternal mobility, relaxation, coping with pain, self-determination and personal comfort result in a higher probability of a VB in hospital, in women with a singleton cephalic presentation at term planning a VB, compared with controls?

Further outcomes to be evaluated will be client-centred outcomes, adverse effects (AEs) and health economic implications [35]. The proposed study replicates the intervention suggested by Hodnett [20], additionally incorporating qualitative findings on the birth environment and birth unit design [14, 21, 24, 25, 27, 36–38] as well as recommendations for the birth environment and positions for labour and birth [31–33, 39].

## Methods/design

### Design

**Fig. 1:**
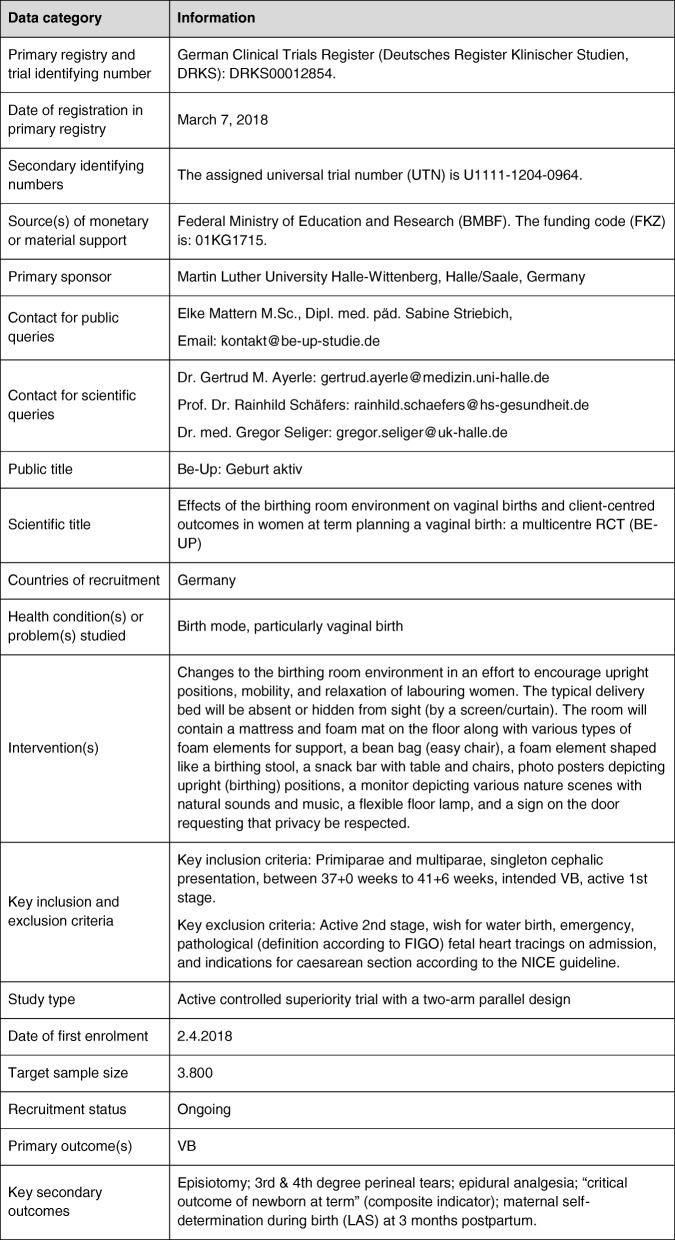
Schedule of enrolment, interventions, and assessments (according to SPIRIT 2013 guidelines [54])

BE-UP (birth environment, upright position) is an active controlled superiority RCT with two study arms (intervention and control group) (see Table 1). The SPIRIT schedule of enrolment, interventions and assessments is provided in Fig. 1 and the SPIRIT checklist in Additional file 1. The trial involves 12 obstetric units (OUs) in hospitals in the German federal states Saxony-Anhalt, Berlin, Brandenburg, Thuringia and North Rhine-Westphalia. Each of the participating OUs handles at least 800 births per year and they have both types of birthing rooms (intervention and control). The study coordination centre for the states Saxony-Anhalt, Berlin, Brandenburg and Thuringia is at the Martin Luther University of Halle-Wittenberg in Halle, Germany; the study coordination centre for the state North Rhine-Westfalia is at the University of Applied Sciences (*Hochschule für Gesundheit*) in Bochum, Germany.

**Table 1:** Design of the BE-UP Study

	Time line							
	Information, confirmation of eligibility, signed consent	Reconfirmation of eligibility, signed consent	Assessment of baseline data (*t*_0_)	Randomisation (when both intervention and control rooms are available)	Intervention/ control	Data assessment points		
					Design of birthing room for women in labour (1st and 2nd stages of labour and birth)	In birthing room (*t*_1_)	Before dis-charge from hospital (*t*_2_)	3 months post-partum (*t*_3_)
Intervention group	Information materials, eligibility log with IC/EC First time: When booking the birth (34th–38th week of pregnancy)	Information materials, eligibility log with IC/EC Second time: On admission to the labour unit (prior to randomisation)	All eligible and consenting participants, prior to randomisation (unbiased)	iPad + WLAN stick for accessing external centrally controlled concealed online randomisation	Intervention birthing room: Special design (reconceptualised)			
					From admission of woman to birthing room up to discharge to postnatal ward or home Emergency equipment available Labour support by midwives and obstetricians			
Controlgroup	Information materials, eligibility log with IC/EC First time: When booking the birth (34th–38th week of pregnancy)	Information materials, eligibility log with IC/EC Second time: On admission to the labour unit (prior to randomisation)	All eligible and consenting participants, prior to randomisation (unbiased)	iPad + WLAN stick for accessing external centrally controlled concealed online randomisation	Control birthing room: Designed according to current practice			
					From admission of woman to birthing room up to discharge to postnatal ward/home; emergency equipment available; labour support by midwives and obstetricians			
Data	Check IC/EC	Check IC/EC	Collect psycho-social and obstetric data		Process data on midwifery and obstetrical care	Assess primary, secondary and other endpoints	Assess experience of birth and breast-feeding and health economic variables	Assess maternal self-determination and health economic variables

*IC/EC* inclusion and exclusion criteria

### Intervention

The experimental intervention will entail a birthing room that has been environmentally re-conceptualised with features and equipment that facilitate mobility, relaxation, coping with pain, self-determination and personal comfort. The typical delivery bed will be absent or hidden from sight (by a screen or curtain). On the floor, there will be a mattress and a foam mat along with various types of foam elements for support, a bean bag (easy chair), a foam element shaped like a birthing stool, a snack bar with a table and chairs, posters depicting upright (birthing) positions, a monitor showing nature films with natural sounds and music, and a flexible floor lamp. There will be a sign on the door requesting that privacy be respected.

This intervention is theoretically based on the sociology of technical artefacts (Linde & Joerges) and Blumer’s symbolic interactionism, both of which stress the significance of the material environment to human behaviour [40]. Each object (e.g. a chair or a bed) with its implicit symbolism and ascribed meaning (for sitting or lying down, respectively) stimulates the behaviour of humans, who in turn use the object in a certain way [41]. The simple presence of (certain) objects changes the structure of an environment, affecting human behaviour and consciousness.

The intervention is, therefore, expected to influence the behaviour of the people in the birthing room. In a room with no delivery bed (or a concealed bed), but with other items, such as a beanbag, a table and chairs, and with an audio-visual presentation, the behaviour of the woman, her partner or companion and staff will be influenced. Using these items will foster an upright position and mobility and will relax the woman, and her individual coping mechanisms will be encouraged. We hypothesise that the physiological processes of labour and birth (e.g. dilation of the cervix, and the descent and rotation of the head) will be supported, and a VB is more likely to be achieved.

The duration of the intervention is from the participant’s admission to the birthing room after randomisation through to birth and discharge or transfer to another ward. Adherence to the study protocol in each OU will be supervised by monitors, who will visit each month, and by lead monitors, who will visit every other month. Each visit will be documented, including any actions undertaken to ensure compliance.

### Control

The control comprises a birthing room in which the delivery bed is centrally located and accessible from three sides. Basically, it does not have any of the additional features and equipment that the intervention room has. However, some of the standard birthing rooms serving as controls also contain a birthing stool, lengths of fabric suspended from the ceiling (used by women in labour for support) or a large exercise ball. For ethical reasons, these birthing rooms will be left as they are, so as not to lower the prevailing standard.

### Participants and eligibility criteria

To be adequately powered, the RCT requires a sample of (about) 3800 women (see Section 2.9). Primiparae and multiparae of all ages with a singleton pregnancy in cephalic presentation, between 37 + 0 weeks to 41 + 6 weeks pregnant on admission to the OU, in active first stage and intending to have a VB are eligible to participate in the study. Pregnant minors will be included if their legal guardian agrees with their participation.

Pregnant women will be *excluded* if they are in the active second stage, want a water birth or have limited ability to understand essential oral and written information about the trial. Women will also be excluded if an evidence-based risk exists for the woman or her baby, i.e. pathological cardiotocography on admission (based on the International Federation of Gynaecology and Obstetrics’s FIGO score), if it is an emergency admission or if there is an indication for CS according to the clinical guidelines of the UK’s National Institute for Health and Care Excellence [39]. The latter includes minor or major placenta praevia, previously diagnosed morbidly adherent placenta, women with human immunodeficiency virus (HIV; under certain conditions), and women with primary genital herpes simplex virus infection occurring in the third trimester of pregnancy.

### Recruitment and randomisation

#### Recruitment

Freelance midwives and obstetricians and gynaecologists practising in the participating hospitals’ catchment areas will be provided with written information (information cards) about the study and asked to hand them out to pregnant women in their care. The participating hospitals will provide pregnant women with written material about the trial and study participation (a brochure and a poster) when they attend hospital antenatal courses or book an appointment at the OU (see Table 1). After confirmation of their eligibility, pregnant women will (again) be informed about the study, orally and in writing, and asked to provide informed consent.

In the PLACE study [20], almost all women approached agreed to participate. In another pilot study (BUMPES trial [42]), a monthly enrolment of 15 women per OU (25% of eligible women) was estimated to be a realistic target. Applying the same calculations to this trial, an enrolment of 3800 women in 12 OUs over the course of 23 months, allowing for the current significantly short staffing in German hospitals, means that each OU would be required to recruit an average of 14 women per month (15.8% of 24,000 eligible women).

#### Randomisation

Only after confirmation of her eligibility and after providing informed consent at admission will a woman be randomised by a midwife or obstetrician and receive an ID code, provided the intervention birthing room and a control birthing room are simultaneously available. Randomisation is ethically acceptable as the birthing environment in both trial groups enables safe midwifery and obstetric care. Individual randomisation is feasible as women usually do not have a choice of which room they will give birth in.

A detailed randomisation plan and lists have been generated by an independent statistician in advance [36], based on the participating OU’s annual baseline rates of VB in 2016. Randomisation will be stratified by units (12 OUs) and parity (two strata, 1:1 per unit). Blinding of either the participants or the attending staff is not possible.

Randomisation will be centrally controlled and concealed. In particular, randomisation will be performed online in real time using a validated dynamic web application provided by the independent Coordination Centre for Clinical Trials (KKS) in Halle (Saale). Each OU will be provided with an iPad with online access to the KKS web application, to ensure that the attending midwife or obstetrician does not need to use the hospital’s electronic devices. After noting the online assignment of the participant to one of the two study groups, the attending midwife or obstetrician will take the woman to the relevant birthing room.

For the rare occasions when the randomisation tool is not available, each OU will also be provided with sealed emergency envelopes containing details of allocation according to the randomisation list prepared by the KKS. The envelopes are kept in a designated study folder in a cupboard that is accessible only to those staff members of the OU who are registered and entitled to carry out randomisation. This ensures that randomisation of study participants is always possible. The midwife or obstetrician carrying out the randomisation is responsible for retrieving the emergency envelope, filling in the forms enclosed in the envelope with the randomisation particulars, signing them and sending them to the KKS by fax. This ensures that KKS receives timely information about the use of an emergency envelope. The KKS will send the unit a replacement emergency envelope by registered mail according to the randomisation list. To prevent tampering, the envelopes must be used in a specified sequence coded by the KKS, the forms containing randomisation particulars must be signed and the emergency envelopes will be regularly checked by the monitors.

### Data collection and outcome measures

The four data assessment points will be on admission (*t*_0_), during labour in the birthing room (*t*_1_), on the postnatal ward before discharge from hospital (*t*_2_), and at 3 months postpartum as a follow-up (*t*_3_) (Table 1). For the follow-up, the participants are asked to provide their contact data and, if possible, the contact data of a second person.

Baseline information (*t*_0_), including psychosocial and obstetric data, will be obtained from eligible and consenting participants prior to randomisation. The baseline data include relevant prognostic factors (independent variables), such as age, body mass index, parity, whether labour is induced and previous CSs. Additionally, the highest educational qualification, migrant background, number of pregnancies, current gestation, any premature rupture of membranes and time of admission to OU will be noted.

The attending midwives and obstetricians will document primary, secondary and other endpoints during active labour (first and second stages) and after the birth. The primary outcome is VB (binary variable), including both spontaneous VB and OVB. Key secondary endpoints, evidence-based and clinically highly relevant, are epidural analgesia, episiotomy, third- and fourth-degree perineal tears (all binary), and maternal self-determination (continuous variable). The latter is the only variable explaining variance in satisfaction with the birth experience [19], and will be assessed by the validated Labour Agentry Scale [37]. Since there is no German version, the 10-item scale will be translated and culturally adapted. It will be validated in a relevant sample of pregnant women living in Germany during the preparation phase of the trial.

Since previous trials used heterogeneous endpoints, an additional key secondary outcome, the composite indicator critical outcome of newborn at term (binary), will be calculated (based on Apgar at 5 min, umbilical artery pH and umbilical artery base excess). Nationally, these outcomes constitute the indicator (ID 1059) for term newborn in a critical condition [1]. Missing data for these outcomes are unlikely, as they constitute routine data.

Other endpoints that will be assessed are the following:

*Binary variables*: Artificial rupture of membranes, oxytocin labour augmentation, use of any analgesia, mode of foetal heart monitoring, prolonged second stage (>30 min or >60 min), blood loss >1000 ml, first- and second-degree perineal tears, other tears to the genital region, maternal transfer to an intensive care unit, maternal death, initiation of breastfeeding, joint discharge of mother and infant from postnatal ward, any diagnosis of health risks in the infant, seeking medical help from a paediatrician or readmission to hospital.

*Nominal variables*: First, second, and third most frequent maternal body positions during labour and birth (as estimated by the midwife), mode of birth, serious adverse events (SAEs; e.g. shoulder dystocia, placental abruption, foetal acidosis, prolapsed chord, HELLP, etc.), use of the delivery bed and breastfeeding.

*Ordinal variables*: Fear of birth (six items [43–45]), adverse events (e.g. pain associated with the perineum, sutures, urination and defecation, breastfeeding, etc.), self-rated health and postnatal depression (Edinburgh Postnatal Depression Scale [38, 46]).

*Continuous variables*: Gestational age, expectations of birth, severe labour pains, experience of birth, subjective usefulness of the equipment in the birthing room, helpfulness of the companion, birth weight, length of stay in the birthing room and on the postnatal ward, infant regulation disorders (relating to crying, nutrition or sleeping) and duration of breastfeeding. Early mother–child bonding will be assessed with the Postpartum Bonding Questionnaire [47],

Additionally, items pertaining to the costs of the intervention and health-care utilisation costs after discharge from hospital (e.g. frequency of medical consultations and use of medication) and money spent on formula or breastfeeding aids will be assessed by a health economic evaluation [35]. Study participants will also be asked to indicate how much they would be willing to pay for an alternative birthing room if they were given the option between a standard or an alternative birthing room.

After conclusion of the recruitment period, midwives and obstetricians will be asked about their experience of and job satisfaction pertaining to the care they provided in the intervention and control birthing rooms (staff-related outcomes).

Variables assessed at *3 months postpartum* include the Labour Agentry Scale, Edinburgh Postnatal Depression Scale, Postpartum Bonding Questionnaire, duration and extent of breastfeeding, maternal and infant health, infant regulation disorders, items pertaining to cost of the intervention and health-care utilisation costs, and willingness to pay. As mothers are generally eager to report on their birth satisfaction postnatally [48], a dropout rate of only 10% at 3 months postpartum [40] is assumed.

### Confidentiality and data security

At first contact (e.g. pregnancy counselling or booking the birth) and on admission to the OU, eligible women will be given oral and written information on the study and their rights in terms of data protection and data handling. The pregnant women will be asked for their written consent regarding (a) participation in the study, (b) confidential handling of their data by authorised members of the study team and (c) use of their contact data for the follow-up data collection at 3 months postpartum. Both parents, or the respective legal guardian, will have to give their consent for the use of the infant’s data. Participants will also be given a further option of providing consent for a secondary analysis of data within the scope of bachelor’s, master’s or doctoral theses. Study participants will receive a copy of the signed informed consent while the original will remain in the OU. All data will be documented in a case report form (CRF), each of which will have a unique code to prevent identification of participants (pseudonymisation) and their allocation (to the trial arms). The list of codes identifying participants will remain in the OU.

Study participants will be informed about their rights under the European General Data Protection Regulation (GDPR), which was enacted on 25 May 2018. Participants will be informed that they can withdraw from the study at any time without giving a reason and with no disadvantage to their care. Data recorded about a participant who withdraws will be deleted at her request.

Access by unauthorised third parties to the CRFs and other data will be prevented by secure handling and storage according to national legal requirements. After the data have been validated in the OU, independent monitors appointed by the sponsor will forward the CRFs to KKS Halle by registered and insured postal delivery. KKS Halle will exclusively hold the list of code numbers and the allocation to the control and intervention groups. After data entry into a data management system and second-look data entry and query management, the paper CRFs will be stored in the medical faculty’s archive for at least 10 years according to legal requirements. After data analysis, the digital data will be securely stored on the server of the Institute of Health and Nursing Science, Martin Luther University of Halle-Wittenberg. Only pseudonymised data will be analysed, and our results will be published in aggregated form.

The lists containing study participants’ contact data will be forwarded by the monitors to the respective trial centres (Martin Luther University of Halle-Wittenberg for the eastern states and the University of Applied Sciences Sciences (*Hochschule für Gesundheit*) in Bochum for North Rhine-Westphalia), where they will be stored securely. The trial centres will use the contact information solely to send questionnaires (and reminders if necessary) to study participants at 3 months postpartum. The contact information sheets will be destroyed immediately after closure of the data collection period unless consent for secondary data analysis was given.

### Data capture and discrepancies

All data will be captured on paper-based CRFs at the OU and transferred to KKS Halle for data management, where the data will promptly be entered into a database. A clinical data management system developed for clinical trials (secuTrial®), which is compliant with good clinical practice, will be used for data entry and query management. All changes in data will be recorded by an audit trail and the data will be saved daily. Data will be electronically checked for plausibility and consistency in a multistage procedure. If there are implausible or missing data, the relevant OU will be asked to obtain any missing information and to resolve inconsistencies. The database is integrated into a general secure system with a firewall and backup system. After completion of data collection, data capture and quality checks, the database will be closed and the data transferred to the biometrician for statistical analysis.

Only after completion of the statistical analyses will the data set be transferred to the trial sponsor, who will keep securely it for 10 years based on national legal requirements.

### Statistical issues and sample size

To determine the rather modest intervention effect of an absolute increase of 5% from an estimated baseline of 74% of VB, the following were noted:

- The rate of CSs in low-risk pregnant women is lower than in all pregnant women, since about one third of CSs are due to multiple births, breech presentations and prematurity or are elective.
- The primary endpoint in this trial is VB, which also includes OVBs.
- In sensitivity analyses, a risk ratio (RR) of 1.20 (95% confidence interval (CI) 1.05 to 1.38; two trials, 240 women) was documented for a spontaneous VB due to increased mobility and upright body positions in the first stage, but there was no statistically significant difference for OVB [6].
- An RR of 1.03 (95% CI 1.01 to 1.05) was found for the likelihood of spontaneous VBs in alternative birth settings, and an RR of 0.89 (95% CI 0.79 to 0.99) for the likelihood of OVBs (8 trials; *n* = 11,202) [14].

As the RR of 1.20 (see (c) above) for a spontaneous VB was calculated based on only two trials with a total of 240 women, we decided on a more conservative increase in the probability of VBs of 5%, i.e. from a baseline of 74% to 79%, corresponding to an RR of 1.07. This takes into account that the increase is more likely to be modest in those OUs where the rate of VBs is already higher. In addition, a smaller effect may be expected due to contamination bias if the same staff care for women in both trial groups.

Using a two-sided Fisher’s exact test, a sample of 3440 women would be required to detect a change of 5 absolute percentage points (from 74% to 79%) in the prevalence of VB (primary outcome) with a power of 90% (a significance level of 5%, assuming a dropout rate of 10%). The sample size has been enlarged to 3800 women to ensure there is sufficient power if there are deviations from the planning assumptions, e.g. increased dropout rates or different baseline rates in OUs recruited to replace study sites that prematurely withdraw from study participation.

### Data analysis

Characteristics of the OUs (centres), study participants and outcomes are described separately for the intervention and control groups (depending on their distribution) using frequency tables, estimated probabilities, 95% CI, means ± standard deviations and percentiles. The analysis performed is intention to treat. In the primary analysis, the probabilities of a VB in the intervention and control groups are compared by Fisher’s exact test using a significance level of 5%. The risk difference and 95% CI are estimated. Additionally, randomised women with missing data in the primary outcome (e.g. dropouts) will be compared to the analysis set with respect to available characteristics, including statistical tests depending on their distribution (Fisher’s test, *t*-test or Wilcoxon rank sum test).

Sensitivity analyses for missing values will be performed in the full intention-to-treat population by multiple imputation, including adjustment for centre effects using logistic regression models with dependent variable VB (yes/no), random effects centre and intervention (versus control) as independent variable (reference for generalised linear mixed models [48]). A further logistic regression model will include an additional random effect of the interaction centre*intervention to adjust for possible centre effects on the intervention effect.

The following variables will be classified and included as independent variables in the logistic regression models above (with and without interaction with intervention) to investigate the role of potential clinically relevant prognostic factors which include: annual baseline rates of VBs of participating OU, age, body mass index, parity, induction of labour with allopathic medicine on admission to birthing room and fear of childbirth.

Five binary, log-normally and ordinally distributed *secondary outcomes* will be compared between the intervention and control groups by Fisher’s test, *t*-test (after a log-transformation if necessary) and Wilcoxon’s test. Multiple testing (five tests) will be adjusted by the Bonferroni method using a reduced significance level of 1%. No imputation is planned for the secondary outcomes.

All tests are two-sided unless otherwise stated. No interim analyses are planned, with the exception of a descriptive analysis to ensure safety (Section 2.11).

#### Subgroup analysis

Subgroup analyses are planned for two parity strata (primiparae and multiparae) and the primary outcome (VB). Subgroup analyses will be performed with respect to the primary outcome using logistic regression analyses. The primary outcome is the dependent variable. Centres are random effects. Independent variables are intervention versus control, subgroup indicators, and the interaction between subgroup and intervention/control.

An additional model including previous CSs as a further independent variable will be fitted in the subpopulation of multiparae only.

The whole analysis will be repeated as a per protocol analysis on the subpopulation after exclusion of participants with protocol violations (expected to be about 10%) or missing values in the primary outcome.

#### Health economics evaluation

As part of the trial, a cost-effectiveness analysis and a cost–benefit analysis will be performed from two perspectives: that of the statutory health insurance and that of the clients (mother and infant) [49]. The analysis to estimate the cost-effectiveness of the intervention in terms of additional costs per additional VB will be performed by calculating an incremental cost-effectiveness ratio, i.e. the ratio of the difference in costs between the intervention group and the control group divided by the difference in the number of VBs. The effect parameter is the primary outcome and will be taken from the trial. The cost–benefit analysis will relate associated costs to participants’ willingness to pay for the use of a redesigned birthing room (such as the intervention room).

Cost parameters will be collected on the postnatal ward before discharge from hospital (*t*_2_) and at 3 months postpartum (*t*_3_). Heath utilisation costs for both mother and baby will be considered, in particular, those related to birth, postpartum health care, hospitalisations (child) and doctor visits (mother and child). Moreover, medication, household help, breastfeeding aids and baby food will be assessed by a questionnaire. Resource use associated with the intervention, i.e. equipment in the birthing room, will be derived from the study documentation. Costs explicitly associated with study conduct, such as data collection or process evaluation, will not be taken into account.

Statistical analyses will be based on intention to treat. Mean differences between intervention and control groups for costs and effects will be calculated. The 95% CIs will be obtained parametrically for the incremental cost-effectiveness ratio according to Fieller’s theorem and non-parametrically by a bootstrap procedure [50]. Univariate and probabilistic sensitivity analyses will be performed to estimate the robustness of the incremental cost-effectiveness ratio. Additionally, a budget impact analysis will estimate the financial consequences for the statutory health insurance in case the programme proves to be cost-effective [51, 52].

All statistical analyses will be done by an independent statistician.

### Benefits, burden and safety

Women in labour will not be able to participate in the study if both birthing rooms required for randomisation are not available. Women with a preference for a particular birthing room will have only a 50% chance of being randomised to it. However, women do not usually get to choose their birthing room. In any case, women will receive midwifery and medical care according to hospital standards. Her partner or companion can be involved in supporting the woman in any birthing room.

There are indications of *benefits* for pregnant women assigned to the *intervention group*:

- The intervention is in accordance with women’s desire for a more home-like environment for their birth. It offers options for various body positions and to move around, allows them to relax and may improve their well-being.
- There is evidence that greater mobility and longer periods of being upright in labour result in a significantly decreased probability of episiotomy and epidural analgesia.
- There is evidence that a VB will result in significantly improved client-centred outcomes, such as maternal satisfaction.

The following *burdens* might be experienced on being assigned to the *intervention group*:

- Continuous foetal heart monitoring may be difficult to achieve in various positions, even if telemetry is used. If necessary, the beanbag, a chair or the mattress could be used for intermittent monitoring or auscultation.

The necessary equipment for women and newborns will be readily available in the event of an emergency. If foetal scalp blood sampling or an OVB become necessary, the screen or curtain can be removed from the birthing bed or the delivery bed can be wheeled into the room.

All AEs and SAEs will be documented in the CRF and validated in 100% of cases, including third- and fourth-degree perineal tears, blood loss >1000 ml, the composite indicator newborn health, serious maternal morbidity (e.g. amniotic fluid embolism) and serious obstetric complications (e.g. shoulder dystocia).

Participants face few foreseeable risks from the intervention. However, to ensure client safety, the following measures are planned. KKS Halle will forward the frequencies of AEs and SAEs at three points during recruitment (when 25%, 50% and 75% of the total sample have been recruited) to the independent data and safety monitoring board (DSMB). This list will detail the incidence *per OU* for the two comparison groups without revealing the identity of the study groups. The DSMB will also compare incidence with national and regional prevalence, and decide on appropriate measures. This process will be documented in writing.

### Involvement of users

Five women’s advocates – representatives from the (i) Federal Initiative to Safeguard Mother and Child during Pregnancy, Birth, and First Year of Life (Mother Hood e.V.), (ii) the Society for Antenatal Preparation, Family Education and Women’s Health (GfG e.V.), (iii) Doulas in Germany (Doulas in Deutschland e.V.) and the (iv) Association of Women’s Health in Medicine, Psychotherapy and Society (AKF e.V.) – have been involved in the preparation of the study. Their role has been to represent the views and concerns of women in labour [53]. They have been consulted on decisions concerning recruitment material, alternative designs of the birthing room, and the participant information sheet and informed consent forms. Two advocates also serve as members of the advisory board. They will receive an allowance to cover their expenses and travel costs, in recognition of their involvement and to maintain their commitment.

### Trial oversight

An independent quality management auditor (QMA-TÜV) will audit the compliance of the OUs with the study protocol in pre-trial visits as required by the funding agency (the Federal Ministry of Education and Research, or BMBF). The auditor, Martina Schlüter-Cruse, is a midwife and research fellow of the University of Applied Sciences, Osnabrück, Germany.

The advisory board has seven members with various backgrounds from Germany and Canada: academic education and research in midwifery, obstetrical clinical practice in hospitals and two women’s advocates. They will advise the study team on challenges and solutions.

The DSMB consists of five professionals from universities in Germany and New Zealand: two clinician/researchers, two researchers (both in midwifery, obstetrics and health care), and one social psychologist with special expertise in statistics. They are independent of the trial team, funding agency and sponsor. At three points in time (when 25%, 50% and 75% of the total sample have been recruited), they will review the rates of AEs and SAEs. The DSMB can discontinue the trial if (after de-blinding) there is a decidedly higher incidence of SAEs (compared with international and national averages) in the intervention group.

## Discussion

This trial is of interest to service users, providers of maternity care, heads of service, academics and researchers. Therefore, the trial and its results will be reported in international peer-reviewed journals, national midwifery and obstetric journals, regional newspapers, and at national and international conferences.

If the intervention proves effective, it can be translated into routine practice at a modest cost and minimal burden to staff. Moreover, nationwide implementation could allow an additional 21,000 women per year to experience a VB instead of a CS. Other expected benefits for women in labour and mothers are increased self-determination during labour, fewer medical interventions, improved client-centred outcomes, fewer CSs in subsequent pregnancies and possibly lower health-care costs.

### Trial status

The RCT was registered with the German Clinical Trials Register (*Deutsches Register Klinischer Studien*) on 7 March 2018, registration number DRKS00012854. The assigned universal trial number is U1111–1204-0964. Additional file 2 lists all items from the World Health Organization Trial Registration Data Set. This protocol is based on version 4 of 7 June 2018. Recruitment of study participants started on 2 April 2018 and will continue until February 2020.

## Additional files

Additional file 1: **Figure S1.** SPIRIT 2013 checklist indicating the location of items addressed in the trial protocol [54]. (PDF 130 kb)

Additional file 2: **Figure S2.** Summary of items from the World Health Organization Trial Registration Data Set. (PDF 129 kb)

## Abbreviations

AE: Adverse event
BE-UP: Birth environment, upright position
CI: Confidence interval
CRF: Case report form
CS: Caesarean section
DSMB: Data and safety monitoring board
HELLP: Hemolysis, elevated liver enzymes, and a low platelet count
KKS: Coordination Centre for Clinical Trials
OU: Obstetric unit
OVB: Operative vaginal birth
RCT: Randomised controlled trial
RR: Relative risk or risk ratio
SAE: Serious adverse event
VB: Vaginal birth

## References

[1] Institut für Qualität und Transparenz im Gesundheitswesen (IQTiG). Bundesauswertung zum Erfassungsjahr 2016 - Geburtshilfe. Berlin: IQTiG - Institut für Qualität und Transparenz im Gesundheitswesen; 2017. https://iqtig.org/downloads/auswertung/2016/16n1gebh/QSKH_16n1-GEBH_2016_BUAW_V02_2017-07-12.pdf. Accessed 23 Oct 2018.

[2] Betran AP, Torloni MR, Zhang J, Ye JF, Mikolajczyk R, Deneux-Tharaux C (2015). What is the optimal rate of caesarean section at population level? A systematic review of ecologic studies. Reprod Health.

[3] Silver RM (2012). Implications of the first cesarean: perinatal and future reproductive health and subsequent cesareans, placentation issues, uterine rupture risk, morbidity, and mortality. Semin Perinatol.

[4] Projektgeschäftsstelle Qualitätssicherung Ärztekammer Sachsen-Anhalt. Jahresauswertung 2016 Geburtshilfe 16/1. 2017. https://www.aeksa.de/files/15DA1A7FB93/2016/st_Gesamt_16n1-GEBH_2016_A8926_2017-5-18_PDFID276584.pdf. Accessed 23 Oct 2018.

[5] QSINDIREKT Nordrhein-Westfalen (2017). Jahresauswertung 2016. Geburtshilfe 16/1.

[6] Lawrence A, Lewis L, Hofmeyr GJ, Styles C. Maternal positions and mobility during first stage labour. Cochrane Db Syst Rev. 2013. 10.1002/14651858.CD003934.pub2. 10.1002/14651858.CD003934.pub4 PMC11664456 24105444

[7] Bodner-Adler B, Bodner K, Kimberger O, Lozanov P, Husslein P, Mayerhofer K (2004). Influence of the birth attendant on maternal and neonatal outcomes during normal vaginal delivery: A comparison between midwife and physician management. Wien Klin Wochenschr.

[8] Ganapathy T (2012). Childbirth in Supported Sitting Maternal Position. International Journal of Nursing Education.

[9] Chang SC, Chou MM, Lin KC, Lin LC, Lin YL, Kuo SC (2011). Effects of a pushing intervention on pain, fatigue and birthing experiences among Taiwanese women during the second stage of labour. Midwifery.

[10] Milligan RA, Parks PL, Kitzman H, Lenz ER (1997). Measuring women's fatigue during the postpartum period. J Nurs Meas.

[11] Dijkstra K, Pieterse M, Pruyn A (2006). Physical environmental stimuli that turn healthcare facilities into healing environments through psychologically mediated effects: systematic review. J Adv Nurs.

[12] Bohren MA, Hofmeyr GJ, Sakala C, Fukuzawa RK, Cuthbert A. Continuous support for women during childbirth. Cochrane Db Syst Rev. 2017. 10.1002/14651858.CD003766.pub6(7). 10.1002/14651858.CD003766.pub6 PMC6483123 28681500

[13] Gupta JK, Hofmeyr GJ, Shehmar M (2012). Position in the second stage of labour for women without epidural anaesthesia. Cochrane Db Syst Rev.

[14] Hodnett ED, Downe S, Walsh D (2012). Alternative versus conventional institutional settings for birth. Cochrane Database Syst Rev.

[15] Kibuka M, Thornton JG. Position in the second stage of labour for women with epidural anaesthesia. Cochrane Db Syst Rev. 2017. 10.1002/14651858.CD008070.pub2. 10.1002/14651858.CD008070.pub3 PMC6464234 28231607

[16] Aburas R, Pati D, Casanova R, Adams NG (2017). The Influence of Nature Stimulus in Enhancing the Birth Experience. Herd-Health Env Res..

[17] Manesh MJ, Kalati M, Hosseini F. Snoezelen Room and Childbirth Outcome: A Randomized Clinical Trial. Iran Red Crescent Me. 2015;17(5). 10.5812/ircmj.17(5)2015.18373. 10.5812/ircmj.17(5)2015.18373 PMC4464378 26082849

[18] Lorentzen I. Clinical Trial: Birth Environment of the Future Clinical Trials 2015 2017. Available from: https://clinicaltrials.gov/show/NCT02478385. Accessed 23 Oct 2018.

[19] Janssen PA, Klein MC, Harris SJ, Soolsma J, Seymour LC (2000). Single room maternity care and client satisfaction. Birth-Iss Perinat C.

[20] Hodnett ED, Stremler R, Weston JA, McKeever P (2009). Re-Conceptualizing the Hospital Labor Room: The PLACE (Pregnant and Laboring in an Ambient Clinical Environment) Pilot Trial. Birth.

[21] Hammond A, Foureur M, Homer CSE (2014). The hardware and software implications of hospital birth room design: A midwifery perspective. Midwifery.

[22] Hammond A, Homer CSE, Foureur M (2017). Friendliness, functionality and freedom: Design characteristics that support midwifery practice in the hospital setting. Midwifery.

[23] Harte JD, Sheehan A, Stewart SC, Foureur M (2016). Childbirth Supporters' Experiences in a Built Hospital Birth Environment: Exploring Inhibiting and Facilitating Factors in Negotiating the Supporter Role. Herd-Health Env Res.

[24] Hauck Y, Rivers C, Doherty K (2008). Women's experiences of using a Snoezelen room during labour in Western Australia. Midwifery.

[25] Hundley VA, Milne JM, Glazener CMA, Mollison J (1997). Satisfaction and the three C's: continuity, choice and control. Women's views from a randomised controlled trial of midwife-led care. Brit J Obstet Gynaec.

[26] Mattern E, Lohmann S, Ayerle G (2017). Experiences and wishes of women regarding systemic aspects of midwifery care in Germany: a qualitative study with focus groups. BMC Pregnancy Childb.

[27] Nilsson C (2014). The delivery room: is it a safe place? A hermeneutic analysis of women's negative birth experiences. Sex Reprod Healthc.

[28] Townsend B, Fenwick J, Thomson V, Foureur M (2016). The birth bed: A qualitative study on the views of midwives regarding the use of the bed in the birth space. Women Birth..

[29] Foureur M, Davis D, Fenwick J, Leap N, Iedema R, Forbes I (2010). The relationship between birth unit design and safe, satisfying birth: Developing a hypothetical model. Midwifery.

[30] Enkin M, Keirse M (2000). A Guide to Effective Care in Pregnancy and Childbirth.

[31] Jenkinson B, Josey N, Kruske S. BirthSpace: An evidence-based guide to birth environment design: Queensland Centre for Mothers & Babies. The University of Queensland; 2013. Available from: https://espace.library.uq.edu.au/view/UQ:339451/UQ339451_fulltext.pdf.

[32] The Royal College of Midwives. Evidence Based Guidelines for Midwifery-Led Care in Labour 2012. https://www.rcm.org.uk/content/evidence-based-guidelines2. Accessed 20 Feb 2018.

[33] Storton S (2007). Step 4: provides the birthing woman with freedom of movement to walk, move, assume positions of her choice: the coalition for improving maternity services. J Perinat Educ.

[34] Bundesministerium für Gesundheit. Nationales Gesundheitsziel: Gesundheit rund um die Geburt. gesundheitsziele.de – Kooperationsverbund zur Weiterentwicklung des nationalen Gesundheitszieleprozesses. Berlin: BMG; 2017. https://www.bundesgesundheitsministerium.de/fileadmin/Dateien/5_Publikationen/Gesundheit/Broschueren/Nationales_Gesundheitsziel_Gesundheit_rund_um_die_Geburt.pdf. Accessed 23 Oct 2018.

[35] Krauth C, Hessel F, Hansmeier T, Wasem J, Seitz R, Schweikert B (2005). Empirical standard costs for health economic evaluation in Germany - a proposal by the working group methods in health economic evaluation. Gesundheitswesen.

[36] Kernan WN, Viscoli CM, Makuch RW, Brass LM, Horwitz RI (1999). Stratified randomization for clinical trials. J Clin Epidemiol.

[37] Hodnett E, Simmonstropea D (1987). The Labor Agentry Scale - Psychometric Properties of an Instrument Measuring Control during Childbirth. Res Nurs Health.

[38] Bergant A, Nguyen T, Heim K, Ulmer H, Dapunt O (1998). Deutschsprachige Fassung und Validierung der ‘Edinburgh postnatal depression scale’. Dtsch Med Wochenschr.

[39] National Institute for Health and Care Excellence (NICE). Intrapartum care for healthy women and babies. 2014 (updated 2017). https://www.nice.org.uk/guidance/cg190/ifp/chapter/Care-of-women-and-their-babies-during-labour-and-birth. Accessed 23 Oct 2018.

[40] Bonß W, Dimbath O (2013). Handlungstheorie: Eine Einführung (Sozialtheorie).

[41] Häußling R (2014). Techniksoziologie.

[42] Brocklehurst P, BUMPES Trial Coordinating Centre. A study of position during the late stages of labour in women with an epidural - The BUMPES study: Protocol 2012. https://www.birmingham.ac.uk/Documents/college-mds/trials/bctu/BUMPES/protocolv5.pdf. Accessed 23 Oct 2018.

[43] Haines H, Pallant JF, Karlstrom A, Hildingsson I (2011). Cross-cultural comparison of levels of childbirth-related fear in an Australian and Swedish sample. Midwifery.

[44] Haines HM, Pallant JF, Fenwick J, Gamble J, Creedy DK, Toohill J, Hildingsson I (2015). Identifying women who are afraid of giving birth: A comparison of the fear of birth scale with the WDEQ-A in a large Australian cohort. Sexual & Reproductive Healthcare.

[45] Ternstrom E, Hildingsson I, Haines H, Rubertsson C (2016). Pregnant women's thoughts when assessing fear of birth on the Fear of Birth Scale. Women Birth.

[46] Cox J, Holden JM, Sagovsky R (1987). Detection of postnatal depression: Development of the 10-item Edinburgh Postnatal Depression Scale. Brit J Psych.

[47] Reck C, Klier CM, Pabst K, Stehle E, Steffenelli U, Struben K (2006). The German version of the Postpartum Bonding Instrument: Psychometric properties and association with postpartum depression. Arch Women Ment Hlth.

[48] Brown H, Prescott R (2006). Applied Mixed Models in Medicine.

[49] Brandes I, Dintsios C-M, Krauth C, Wasem J (2005). Die Perspektive der Gesetzlichen Krankenversicherung in der gesundheitsökonomischen Evaluation. Z gesamte Versicherungswissenschaft.

[50] Drummond M, Sculpher M, Claxton K, Stoddart G, Torrance G (2015). Methods for the economic evaluation of health care programmes.

[51] Mauskopf JA, Earnshaw S, Mullins CD (2005). Budget impact analysis: review of the state of the art. Expert Rev Pharmacoecon Outcomes Res.

[52] Sullivan SD, Mauskopf JA, Augustovski F, Jaime Caro J, Lee KM, Minchin M (2014). Budgetimpact analysis-principles of good practice: report of the ISPOR 2012 Budget Impact Analysis Good Practice II Task Force. Value Health.

[53] Boote J, Barber R, Cooper C (2006). Principles and indicators of successful consumer involvement in NHS research: Results of a Delphi study and subgroup analysis. Health Policy.

[54] Chan A-W, Tetzlaff JM, Altman DG, Laupacis A, Gøtzsche PC, Krleža-Jerić K, Hróbjartsson A, Mann H, Dickersin K, Berlin J, Doré C, Parulekar W, Summerskill W, Groves T, Schulz K, Sox H, Rockhold FW, Rennie D, Moher D (2013). SPIRIT 2013 Statement: Defining standard protocol items for clinical trials. Ann Intern Med.

