# Supplementary material for: Effects of the birthing room environment on vaginal births and client-centred outcomes for women at term planning a vaginal birth: BE-UP, a multicentre randomised controlled trial
Source: Trials. 2018 Nov 19;19:641. doi: 10.1186/s13063-018-2979-7 (PMC6245933; doi:10.1186/s13063-018-2979-7)
Supplement: Supplementary file 2 — Figure S2. Summary of items from the World Health Organization Trial Registration Data Set. (PDF 129 kb) [file 13063_2018_2979_MOESM2_ESM.pdf]

	Enrol-ment	Allo-cation				Close-out
**TIMEPOINTS:**	**-*t*₁** *(before/ on admission)*	**0** *(on admission)*	***t*₁** *(during labour and birth)*	***t*₂** *(on post-natal unit)*	***t*₃** *(3 months post-partum)*	***t*₄** *(after con-clusion of recruit-ment)*
**ENROLMENT:**						
**Eligibility screen**	X					
**Informed consent**	X					
**Allocation**		X				
**INTERVENTIONS:**						
Redesigned birthing room (intervention)			X			
Conventional birthing room (control)			X			
**ASSESSMENTS:**						
Inclusion & exclusion criteria	X	X				
<u>Sociodemographic data</u>: health insurance, highest school education, single mother without partner support, migrant background		X				
<u>Prognostic factors</u>: age, BMI, gravidity, parity, induction of labour, previous CS; Additionally: week of gestation, premature rupture of membranes, and time of admission to OU.		X				
<u>Primary outcome</u>: vaginal birth <u>Secondary outcomes</u>: epidural analgesia, episiotomy, 3rd & 4th degree perineal tears, blood loss >1,000ml, "critical outcome of newborn at term"			X			

– continued on next page –

	Enrol-ment	Allo-cation				Close-out
**TIMEPOINTS:**	**-t_1** *(before/ on admission)*	**0** *(on admission)*	**t_1** *(during labour and birth)*	**t_2** *(on post-natal unit)*	**t_3** *(3 months post-partum)*	**t_4** *(after con-clusion of recruit-ment)*
***Other endpoints**: artificial rupture of membranes, oxytocin labour augmentation, mode of fetal heart tracing, use of any analgesia, maternal body positions during labour and birth, use of the birthing bed; prolonged second stage (> 30 min, > 60 min), mode of birth, 1st and 2nd degree perineal tears, other genital tears, serious adverse events (e.g. shoulder dystocia, placental abruption, fetal acidosis, prolapsed chord, HELLP, etc.); newborn: time of birth, sex, birth weight.*				X		
*Maternal transfer to postnatal unit/ICU, joint discharge of mother and infant from postnatal unit, maternal death; **Participant-reported data**: expectations of birth, fear of birth (WDEQ-A), subjective experience of labour pain, of usefulness of equipment in birthing room, of positions and mobility, of staff and support persons, initiation and duration of breastfeeding*				X		
***Participant-reported data**: Self-rated maternal health, physical/emotional wellbeing, expectations of birth, maternal self-determination (LAS), postnatal depression (EPDS) adverse effects (e.g. pain associated with the perineum, sutures, urination and defecation, breastfeeding, etc.), stress due to infant regulation disorders (crying, nutrition, sleeping), early mother-child bonding (PBQ), transfer of newborn to other unit/hospital/ICU, documented health risks of infant, readmission to hospital, out-of-hospital medical treatment of mother or newborn, pain medication or antibiotics for mother or newborn, domestic health/family care worker, medical aids, amount and duration of breastfeeding, cost of formula fed, willingness to pay for the alternative birthing room (intervention) in a future birth*					X	
***Staff-reported data:** job satisfaction of midwives and obstetricians*						X
